# The relationship between maternal age, body mass index, and the rate of preterm birth

**DOI:** 10.4274/jtgga.2018.0057

**Published:** 2018-11-15

**Authors:** Joachim W. Dudenhausen, Mirjam Kunze, Ursula Wittwer-Backofen, Hans Peter Hagenah, Alexander Strauss, Veronika Günther, İbrahim Alkatout, Amos Grunebaum, Manfred Voigt

**Affiliations:** 1 Department of Obstetrics, Charité University School of Medicine, Berlin, Germany; 2 Department of Gynecology and Obstetrics, University of Freiburg School of Medicine, Freiburg, Germany; 3 Center of Medicine and Society, University of Freiburg, Freiburg, Germany; 4 Department of Obstetrics, Agaplesion Diakonieklinikum Rotenburg, Rotenburg, Germany; 5 University of Kiel, Kiel, Germany; 6 Department of Gynecology and Obstetrics, University Hospitals Schleswig-Holstein, Kiel, Germany; 7 Department of Gynecology and Obstetrics, Weill Cornell Medicine, New York, USA; 8 University of Freiburg School of Medicine, Freiburg, Germany

**Keywords:** Maternal age, obesity, body mass index, premature birth, Germany

## Abstract

**Objective:**

The aim of the present study was to assess the influence of maternal age and maternal body mass index of early pregnancy on the risk of preterm delivery.

**Material and Methods:**

The study included 2.1 million liveborn single newborns with documented data at perinatal surveys. Statistical analyses were performed using the SPSS statistics program.

**Results:**

The risk of preterm births increased in obese women and in women with advanced age.

**Conclusion:**

Strategies should be developed to reduce preconceptional body mass index, and guidelines are required to help advise women who postponed childbearing.

## Introduction

Over the past decades, there has been a significant increase in the average age among primipara women, and a rise in the body mass index (BMI) among pregnancies in high-income countries. 

In 2011, the average age of the mother at the birth of her first child in Germany was 29.1 years, and overall, the average age of women at childbirth was 30.6 years. 

**Figure 1:**
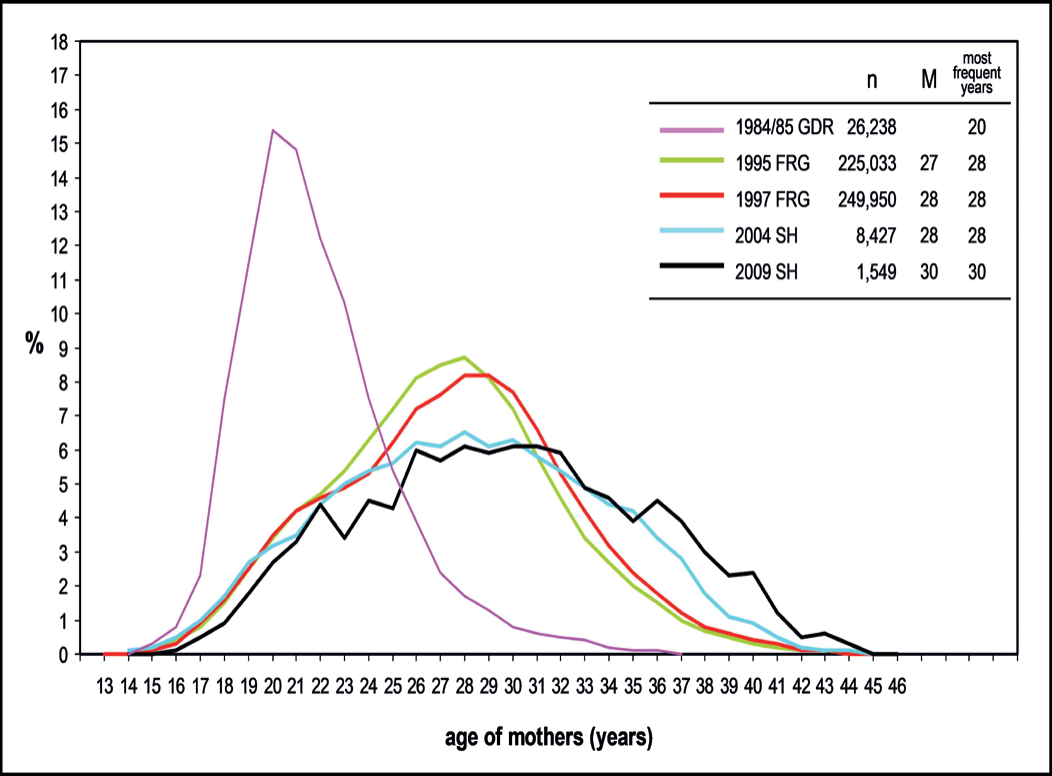
Distribution (%) of maternal age at the time of first pregnancy in Germany *GDR: German Democratic Republic; FRG: Federal Republic of Germany; SH: Schleswig-Holstein*

Figure 1 shows the development of the age distribution of nulliparous women in the Federal Republic of Germany, Schleswig-Holstein and the German Democratic Republic, during the various years. Regional differences can be interpreted by social, political, and medical developments.

In the United States, according to the National Center for Health Statistics (1), the birth rate of 40 to 44-year-olds has doubled between 1981 and 2003. Many publications have shown that late maternity is associated with various risks to the mother and various risks to perinatal outcomes, such as preterm delivery (PTD), and chromosomal aberrations (2,3,4). Women are generally well informed about age-related decreasing fertility rates and the increasing risk of trisomy 21, but they are not as well informed about pregnancy-related risks based on increased maternal age (5). Controlled clinical studies and evidence-based guidelines for advising women who postpone childbearing are necessary (6).

In addition to an increasing maternal age, an increase in maternal weight could also be observed (7). The most commonly used measurement for defining obesity is BMI, which refers to an individual’s weight in kilograms divided by the square of their height in meters (kg/m^2^).

The consequences of the increase in maternal weight are significant for all health systems. For example, it is important to assess the impact of the mother’s age-related weight increase on prematurity rates and fetal and neonatal outcomes.

## Material and Methods

The World Health Organization and the Institute of Medicine define a BMI of under 18.5 kg/m^2^ as underweight, from 18.5 to 24.9 kg/m^2^ as a normal and healthy weight, from 25.0 to 29.9 kg/m^2^ as overweight, and above 29.9 kg/m^2^ as obesity. Within the obesity category, a further division into three can be made: a BMI from 30.0 to 34.9 kg/m^2^ can be defined as obesity grade I, from 35.0 to 39.9 kg/m^2^ as obesity grade II, and a BMI ≥40.0 kg/m^2^ as obesity grade III (8).

This was a retrospective study that included singleton women who delivered in Germany. The data analyzed in the paper were obtained from the routine data collection undertaken by the German Perinatal Survey, a mandatory survey conducted throughout Germany. The data included singleton pregnancies from 1984/85 to 2008/2009 for Germany and the federal state Schleswig-Holstein. From 1995 to 1997, the state of Baden-Württemberg was excluded; from 1998 to 2000 the states of Baden-Württemberg, Berlin, Hesse, North Rhine-Westphalia, Rhineland-Palatine, Saarland, Schleswig-Holstein, were excluded. The inclusion and exclusion criteria are based on whether the individual federal states submitted their data with regard to the perinatal survey. The exclusion of a federal state is therefore due to non-existent data by the federal state itself. The study population consisted of a total of 2,130,594 pregnancies with liveborn infants.

The women’s BMI was classified following the recommendations of the Institute of Medicine of the United States. PTD was defined as those <37 gestational weeks.

The data were analyzed using descriptive statistics. The data center of the University of Rostock performed the statistical analysis using the SPSS computer program, version 22.0.

## Results

**Figure 2:**
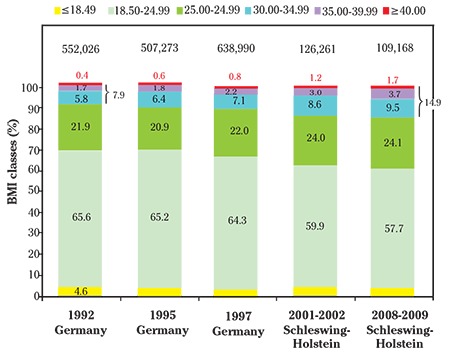
Trends in body mass index of mothers who delivered in Germany (between 1992 and 1997) and Schleswig-Holstein (between 2001 and 2009) *BMI: Body mass index*

Between 1992 and 2009, there was a decrease in the rate normal pre-pregnancy BMI from 65.6% (362,419/552,026) to 57.8% (10,184/17,621), as well as an increase of obesity I-III from 7.9% (43,440/552,026) to 14.9% (2,719/17,621) (Figure 2). For 1992 to 1997, the data from all over Germany are shown, for the period 2001 to 2009, the data from Schleswig-Holstein were used as an example.

**Figure 3:**
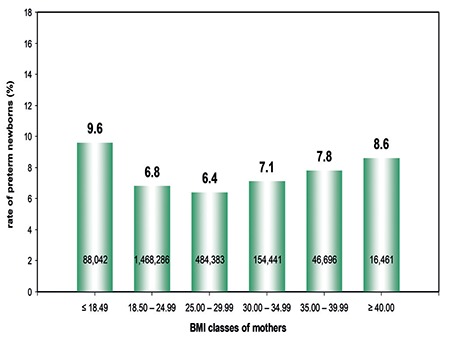
Rate of preterm newborns (%) in relation to the maternal body mass index (Germany, 1995-2000, n=2,258,309) *BMI: Body mass index*

The PTD rate in those with a normal BMI was lower (6.8%; 99,918/1,468,286) as compared with 8.6% (1,423/16,461) in the group of obese women (III) (Figure 3). On the other hand, mothers with BMI ≤18.49 kg/m^2^ had the highest risk of premature birth (9.6%).

Mothers with a BMI between 25.00 and 29.99 kg/m^2^ had the lowest risk of premature birth (6.4%). Subsequently, the risk of premature birth increased gradually with increasing BMI and reached its maximum in mothers of obesity group III with 8.6%.

**Figure 4:**
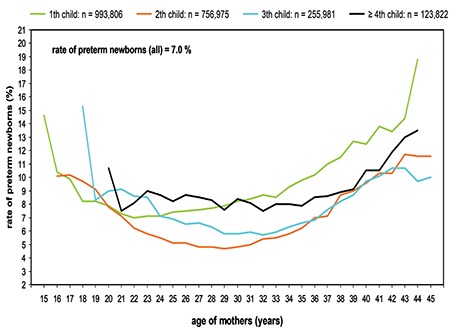
Rate of preterm birth as a function of maternal age and parity (Germany, 1995-2000, n=2,130,584)

There were clear differences of the PTD rates related to maternal age and parity (Figure 4). Whether it was the first, second, third or fourth child - all curves showed a U-shaped course, which shows that the risk of premature birth was increased in both younger and older mothers. The lowest rates of premature birth can be observed in second-born children. At a maternal age of 16 years, the premature birth rate was 10%, then dropped to a low of 5% at a maternal age of 29 years, and then rose again. At a maternal age of 45 years, the premature birth rate was 11%. After second-born children, the third-born had the lowest rates of premature birth. Individual outliers of the curve can be explained by the low number of cases (e.g. third child at the maternal age of 19 years). This curve runs approximately parallel to the first one.

The premature birth rate in first-born children showed a steeper increase than the three other curves from a maternal age of 33 years.

The premature birth rate in the fourth child and subsequent children was relatively constant at 8.5% and only increased from a maternal age of 39 years (maximum at 44 years: premature birth rate of 13%).

**Figure 5:**
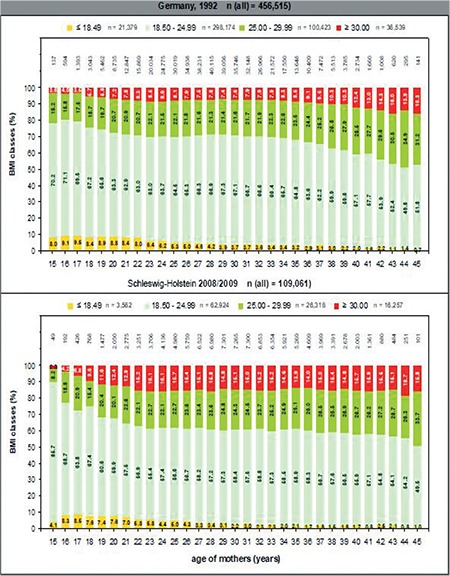
Distribution of body mass index at maternal age in Germany 1992 and Schleswig-Holstein 2008/2009 *BMI: Body mass index*

Figure 5 shows the distribution of the BMI groups in the various years of childbirth in Germany in 1992 versus in Schleswig-Holstein in 2008/9. The proportion of 23-year-old women with normal BMI in early pregnancy was 63% in 1992 and 54.9% in 2008/9.

## Discussion

Our data show an increase in obesity I-III, as well as a decrease in the rate of normal pre-pregnancy BMI between 1992 and 2009. The risk of PTD increases with the maternal BMI and shows a risk of 8.6% in obesity group III. Our results confirm the effect a mother’s age has on the PTD rates. Especially as it relates to parity, this relationship is biphasic: the premature delivery rate is high at both ends of a woman’s age, for young women (under 20 years) and also for older women. Many authors have demonstrated the increased risk of premature birth in younger people (under 18 years) and especially in older women (9,10). The lowest prematurity rates are found in the following age groups, depending on the parity: for the first child, the premature birth rates are lowest in the age range of 21 to 24 years, for the second child between the ages of 25 and 31 years, for the third child between 28 and 34 years, and from the 4^th^ child on between 29 and 35 years (Figure 4). Therefore, the optimal age for a pregnancy with regard to the prematurity rate can only be determined from the parity aspect: for a woman with her 1^st^ child, this ‘optimal’ age section is earlier than for women who have their 2^nd^, 3^rd^ or 4^th^ child.

Frederiksen et al. (11) investigated the relationship between advanced maternal age and unfavorable outcomes of pregnancy. Approximately 370,000 single pregnancies were included between 2008 and 2014. Pregnant women of advanced age were divided into two groups: 35-39 years and 40 years and older. The comparison group was formed by pregnant women aged 20-34 years. Pregnancies were followed from the end of the first trimester until birth. The primary endpoint was the occurrence of an unfavorable outcome, such as chromosomal abnormalities, congenital malformations, stillbirth and premature birth before the 34^th^ SSW. The researchers concluded that pregnant women aged over 40 years had a significantly higher risk of chromosomal abnormalities (increased 7.4 times), miscarriages (increased 3.1 times), and premature births before the 34^th^ SSW (increased 1.7 times) compared with women aged 20-34 years. These results must be taken into account during prenatal care and this risk group must be monitored more closely. These results from Denmark are congruent to the results regarding the maternal age in our study.

A study from Sweden (12) investigated the relationship between maternal obesity and the risk of premature birth. A total of 1,599,551 deliveries were examined between 1992 and 2010. Preterm deliveries were divided into three groups: (extremely premature, 22-27 weeks; very premature, 28-31 weeks and moderately premature, 32-36 weeks). Risks of extremely, very, and moderately preterm deliveries increased with BMI and the overweight and obesity-related risks were highest for extremely preterm deliveries. Among normal-weight women (BMI 18.5 to <25 kg/m^2^), the rate of extremely PTD was 0.17%. As compared with normal-weight women, rates (%) and adjusted odds ratios [ORs (95% CI)] of extremely PTD were as follows: BMI 25 to <30 <(0.21%; OR, 1.26; 95% CI: 1.15-1.37), BMI 30 to <35 (0.27%; OR, 1.58; 95% CI: 1.39-1.79), BMI 35 to <40 (0.35%; OR, 2.01; 95% CI: 1.66-2.45), and BMI of >40 or greater (0.52%; OR, 2.99; 95% CI: 2.28-3.92). Risk of spontaneous extremely PTD increased with BMI among obese women (BMI ≥30 kg/m^2^).

In Sweden, as well as in our study, maternal overweight and obesity during pregnancy were associated with increased risks of PTD, especially extremely PTD.

Cleary-Goldman et al. (13) confirmed the influence of maternal age on the rate of preterm deliveries in around 36,000 single deliveries, that increased age of the mother was an independent risk factor for pregnancy outcomes such as gestational diabetes and macrosomia. This was confirmed by Abu Hamad et al. (14), by Kenny et al. (15) for the influence of the socio-economic status, and by Baer et al. (16) regarding the influence of ethnicity.

This development is partly due to the use of artificial reproductive medicine (17), but also by the extension of the education period, the later entry into the working life, later marriages and longer phases of the partner search, the argument of the loss of work or the threat to the career of the employed woman, which all lead to delayed childbirth and family planning with increasingly shifted higher age in many women. Whether ‘social freezing’ has had a statistical impact in recent years is unclear. In the future, however, one would expect a further shift in the gestational age, which will then be associated with significantly higher premature infants and possibly other risks for maternal and fetal outcome.

According to the Federal Statistical Office, 52% of the adult population in Germany was overweight in 2013 (62% of men and 43% of women). In comparison to 1999, the proportion of overweight adults has risen (48%, 56% of men and 40% of women). A total of 16% were obese in Germany (17% of men and 14% of women) (18).

In perinatal data from 1989 to 2000 in Germany, Briese et al. (19) showed a significantly increased 3.3-fold rate of severe hypertrophic neonates among morbidly obese women when compared with normal weight women. The rate of complications such as preeclampsia, gestational diabetes, and neonatal infections and hyperbilirubinemia were significantly more frequent. The rate of caesarean delivery among women with a BMI over 45 kg/m^2^ was 38% as compared with normal weight women with 18%.

Future increasing maternal age and increasing pre-pregnancy BMI suggest a further future rise in PTD rates in high-income countries. Premature delivery is often associated with fetal risks, such as respiratory adaptation disorders, temperature regulation or aggravated food intake. Preventive preconception strategies for reductions of pre-pregnancy BMI of overweight and obese women and guidelines for counselling women who plan to postpone their wishes for children to later life are urgently needed.
